# Correlative super-resolution fluorescence and electron microscopy using conventional fluorescent proteins *in vacuo*

**DOI:** 10.1016/j.jsb.2017.05.013

**Published:** 2017-08

**Authors:** Christopher J. Peddie, Marie-Charlotte Domart, Xenia Snetkov, Peter O'Toole, Banafshe Larijani, Michael Way, Susan Cox, Lucy M. Collinson

**Affiliations:** a Electron Microscopy STP, The Francis Crick Institute, 1 Midland Road, London NW1 1AT, UK; b Cellular Signalling and Cytoskeletal Function, The Francis Crick Institute, 1 Midland Road, London NW1 1AT, UK; c Department of Biology, The University of York, Heslington, York, UK; d Cell Biophysics Laboratory, Ikerbasque, Basque Foundation for Science, Research Centre for Experimental Marine Biology and Biotechnology (PiE), Biofisika Institute (CSIC, UPV/EHU) and, University of the Basque Country (UPV/EHU), Leioa 48940, Spain; e Randall Division of Cell and Molecular Biophysics, New Hunt’s House, King’s College London, London SE1 1UL, UK

**Keywords:** CLEM, ILSEM, Correlative, Volume, Electron microscopy, Super-resolution, Integrated, YFP, GFP, Fluorescence, Protein localisation, 3-dimensional, Blinking, In-resin fluorescence

## Abstract

Super-resolution light microscopy, correlative light and electron microscopy, and volume electron microscopy are revolutionising the way in which biological samples are examined and understood. Here, we combine these approaches to deliver super-accurate correlation of fluorescent proteins to cellular structures. We show that YFP and GFP have enhanced blinking properties when embedded in acrylic resin and imaged under partial vacuum, enabling *in vacuo* single molecule localisation microscopy. In conventional section-based correlative microscopy experiments, the specimen must be moved between imaging systems and/or further manipulated for optimal viewing. These steps can introduce undesirable alterations in the specimen, and complicate correlation between imaging modalities. We avoided these issues by using a scanning electron microscope with integrated optical microscope to acquire both localisation and electron microscopy images, which could then be precisely correlated. Collecting data from ultrathin sections also improved the axial resolution and signal-to-noise ratio of the raw localisation microscopy data. Expanding data collection across an array of sections will allow 3-dimensional correlation over unprecedented volumes. The performance of this technique is demonstrated on vaccinia virus (with YFP) and diacylglycerol in cellular membranes (with GFP).

## 1 Introduction

Life science imaging techniques have seen a number of revolutionary advances in recent years. Super-resolution light microscopy now allows visualisation at resolutions beyond the diffraction limit of light, and new electron imaging systems based on the scanning electron microscope (SEM; such as FIB SEM, SBF SEM, and array tomography) can deliver 3-dimensional cell and tissue ultrastructure through unprecedented volumes (Peddie and Collinson, 2014). At the same time, the practical application of correlative workflows that directly link protein localisation to cell ultrastructure has increased rapidly. Examples of protocols that correlate super-resolution light and electron microscopy from separate instruments include photoactivated localisation microscopy (PALM) with TEM and SEM (Betzig et al., 2006, Chang et al., 2014, Kopek et al., 2013, Sochacki et al., 2014, Watanabe et al., 2011), and stochastic optical reconstruction microscopy (STORM) with SEM (Loschberger et al., 2014, Wojcik et al., 2015). However, combining data from separate imaging systems can limit interpretation, as many techniques rely on the use of specific imaging conditions and probes that are not necessarily compatible with high precision correlation to electron microscopy data.

Probes are a key component in super-resolution correlative imaging. The GFP family, a ubiquitous tool in bioscience research, can be preserved through processing into the resin polymers that are necessary to support the specimen during electron imaging *in vacuo* (Bell et al., 2013, Johnson et al., 2015, Kukulski et al., 2011, Nixon et al., 2009, Peddie et al., 2014, Watanabe et al., 2011). We recently showed that YFP and GFP are photostable when embedded in acrylic resins using an in-resin fluorescence (IRF) protocol. Conservation of the hydration state of the FPs was critical for stable, long-lived fluorescence, and also for imaging ultrathin IRF sections *in vacuo* using an integrated light and scanning electron microscope (ILSEM) (Brama et al., 2015, Peddie et al., 2014). Fluorescence and electron images of ultrathin IRF sections are unique in that the information from both imaging modalities is gathered from the same physical slice (50–200 nm thick). As such, the axial (z) resolution corresponds to the physical slice thickness and the fluorescence images taken from IRF sections are inherently better than the diffraction limit. However, the lateral (xy) plane will still be diffraction limited if a widefield fluorescence (WF) microscope is used, so structures below ∼200 nm cannot be resolved. Intriguingly, it was recently shown that standard FPs blink in IRF sections when imaged using a customised widefield fluorescence microscope and glycerol based mountant prior to imaging in a TEM (Johnson et al., 2015), or using STORM with a reducing buffer prior to imaging in a SEM (Kim et al., 2015). Here, we show that FPs also blink in dry mounted IRF sections, enabling *in vacuo* single molecule localisation and high-precision correlation of FP-labelled macromolecules to cell ultrastructure.

We illustrate the potential of this new high-precision correlative technique using HeLa cells infected with YFP-A3 Vaccinia virus (Arakawa et al., 2007) and HeLa cells transfected with GFP-C1. Viral particles are easily recognised based on morphology in electron micrographs; early immature virions (IV) are spherical and typically 280 nm in diameter, while mature virions (IMV, IEV) develop into brick shapes of 360 nm in the largest dimension (Condit et al., 2006, Cyrklaff et al., 2005, Sodeik and Krijnse-Locker, 2002). When labelled with YFP-A3, a core viral protein (Wilton et al., 1995), the fluorescent signal is punctate (Arakawa et al., 2007, Horsington et al., 2012). Viral particles in electron micrographs can therefore be used to assess the precision with which FPs can be located to the underlying structure (Kukulski et al., 2011). GFP-C1, which targets the lipid DAG at the endoplasmic reticulum, nuclear envelope, nucleoplasmic reticulum, and Golgi apparatus (Domart et al., 2012, Peddie et al., 2014), demonstrates that another FP from the same family in a different subcellular environment blinks under the same conditions, paving the way for multi-colour super-resolution CLEM.

## 2 Results

### 2.1 GFP and YFP blink in fixed whole cells at atmospheric pressure

**Fig. 1:**
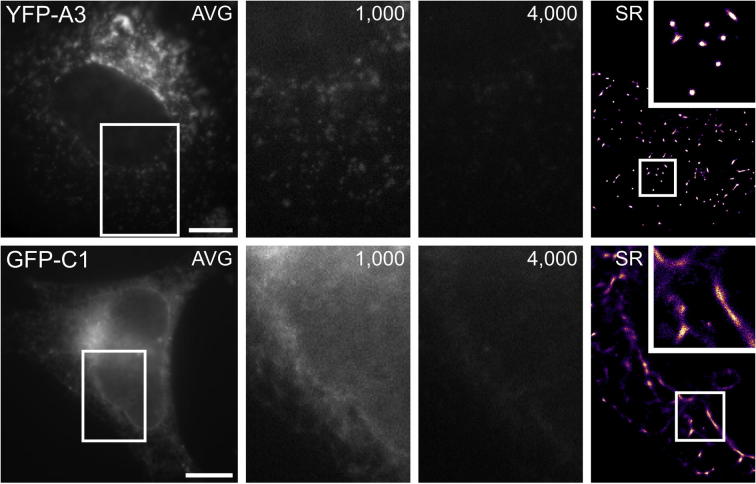
FP blinking in fixed HeLa cells at atmospheric pressure using the SECOM stage. Shown are average intensity projections through the image stack (AVG; generated from frames 500–4,000), individual images at 1,000 and 4,000 frames elapsed, and a ThunderSTORM generated reconstruction of the image stack with higher magnification insert. For YFP-A3 vaccinia, the punctate localisation expected from a viral core protein was present in areas of lower fluorophore density e.g. the cell periphery. Localisation of GFP-C1 to the putative Golgi apparatus was visible, in addition to a clearly outlined nuclear envelope. Scale bars – 10 µm (YFP-A3), 5 µm (GFP-C1).

To provide a baseline readout of FP blinking, we first imaged YFP-A3 vaccinia and GFP-C1 in whole fully-hydrated HeLa cells that were fixed in 4% PFA and mounted in Citifluor AF4 using the SECOM light microscope at atmospheric pressure in WF mode (Fig. 1). Both YFP and GFP displayed blinking behaviour when the laser power was increased to a density of 330 W/cm^2^, and progressive photobleaching was apparent in the samples over the course of an image acquisition series of 4,000 frames (Fig. 1, supplementary movies 1 and 2). However, a substantial proportion of blinking molecules were obscured by out of focus fluorescence, which may lead to localisation errors. Super-resolution (SR) image reconstruction was carried out using ThunderSTORM (Ovesny et al., 2014). YFP-A3 localisation was punctate, as expected for individual virus particles (Fig. 1, upper panel). GFP-C1 was observed at the nuclear envelope as previously reported for DAG localisation (Fig. 1, lower panel), and in other cytosolic locations that could not be assigned to specific cellular organelles by fluorescence microscopy alone.

### 2.2 Workflow for SR CLEM *in vacuo* in the ILSEM

**Fig. 2:**
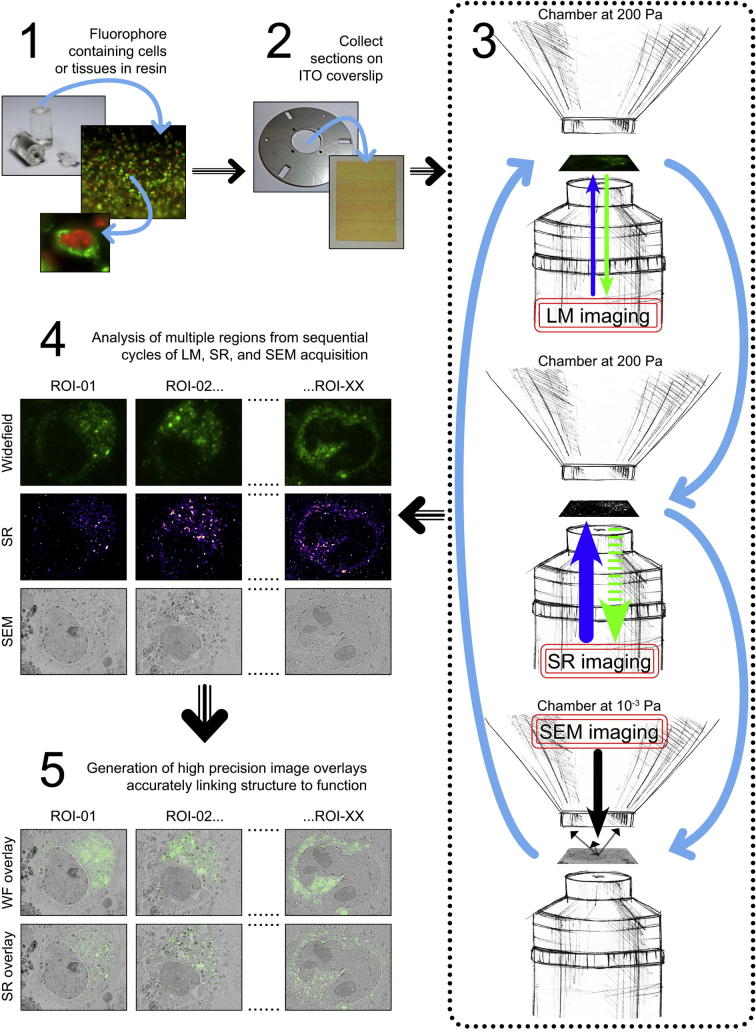
Schematic illustrating the typical workflow for WF, SR and EM imaging (modified from Brama et al. (2015)). Specimens were first processed for high pressure freezing and quick freeze substitution as described in Peddie et al. (2014), embedded in acrylic resin, and collected as 200 nm serial sections on an ITO-coated glass coverslip. The coverslip was attached to a SECOM specimen holder, and placed on the SECOM microscope stage. The SEM chamber was pumped to 200 Pa, and WF and SR image acquisition for a specific region of interest was carried out before pumping to high vacuum for SEM BSE image acquisition; after which, the vacuum pressure could be cycled to allow for multiple rounds of image acquisition from several regions of interest within the same specimen, or across serial sections using an array tomography workflow.

HeLa cells infected with YFP-A3 vaccinia or transfected with GFP-C1 were high pressure frozen, freeze substituted and embedded in HM20 resin as previously described (Peddie et al., 2014) (Fig. 2, step 1). Thin sections of ∼ 200 nm were cut from the resin block (Fig. 2, step 2). Fluorescent cells were located using the SECOM light microscope in WF mode within the SEM at 200 Pa partial pressure of water vapour, which we previously demonstrated to be optimal for WF imaging *in vacuo* (Brama et al., 2015). The laser power was increased to a density of 330 W/cm^2^, to drive FP blinking, and sequences of ∼30,000 images were collected. The vacuum pressure was then decreased to 10^−3^ Pa (high vacuum) for optimal backscattered electron imaging. This WF-SR-EM cycle could be repeated sequentially to image different cells in the same section, or the same cell across serial sections to build up a 3D volume (Fig. 2, step 3). The SR images were reconstructed using ThunderSTORM to generate individual localisations (Fig. 2, step 4). Lastly, overlays of WF-EM and SR-EM were created to identify fluorescently labelled cellular structures (Fig. 2, step 5).

### 2.3 YFP-A3 blinks *in vacuo*, enabling SR CLEM localisation of YFP-A3 to the core of vaccinia virus

**Fig. 3:**
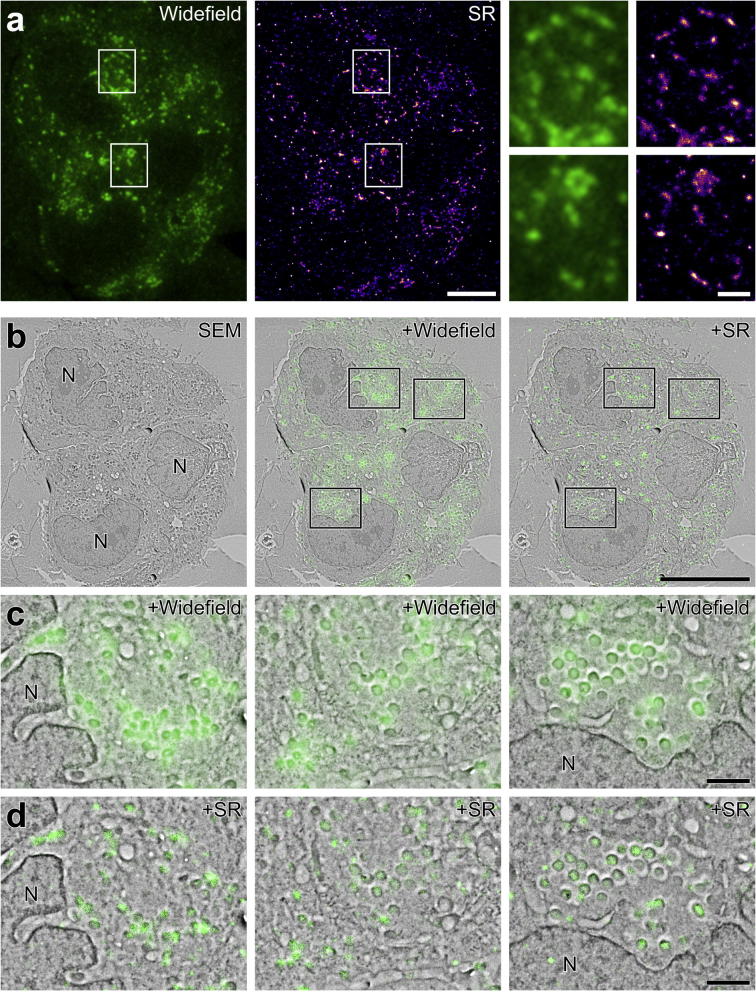
Comparison of WF to SR highlights significantly improved lateral resolution: YFP-A3. a: WF image showing localisation of YFP-A3 vaccinia within a HeLa cell. The SR reconstruction from a sequence of approximately 30,000 images represents 728,076 individual localisations. Enlargements of the boxed areas show the improved precision of YFP-A3 localisation from SR based image reconstruction. b: Micrograph showing the BSE signal from the group of three cells in A with WF image and SR reconstruction overlaid. c, d: Enlargements of the boxed areas in B showing localisation of the YFP signal to groups of virus particles. Vaccinia virus protein A3 localises to the inner core wall of developing virions (Roos et al., 1996, Wilton et al., 1995). As such, whilst overlay of the WF image highlights closely apposed groups of virions within the cytoplasm, overlay of the SR reconstruction allows more precise identification of specific virions expressing YFP-A3. N: nucleus. Scale bars - 5 µm (a), 10 µm (b), 1 µm (A inset, c, d).

Reconstructed SR images of YFP-A3 vaccinia-infected HeLa cells in IRF sections *in vacuo* were of higher resolution than WF images of the same section (Fig. 3a; SMovie 3). Resolution was measured using Fourier Ring Correlation (FRC) (Nieuwenhuizen et al., 2013) to be 85.5 ± 13.1 nm, assessed using 10 patches of size 2.7 μm^2^ across the image and twenty statistical repeats. Only patches that contained a significant amount of data were used in the evaluation since there were substantial areas of the image with very low information content. While this resolution was slightly lower than that typically achieved using organic dyes (e.g. 60 nm for tubulin labelled with Alexa 647 in Nieuwenhuizen et al. (2013)), this was unsurprising as the photon yields of FPs are typically lower. After WF and SR image acquisition, EM images were acquired in high vacuum (Fig. 3b). Composite images were generated to demonstrate the precision of correlation between WF-EM and SR-EM (Fig. 3c). The punctate distribution of YFP-A3 and the recognisable ultrastructure of the virus particles aided the alignment of light and electron images. YFP-A3 localised to viral particles from a range of developmental stages (Fig. 3c and d). Though the lateral resolution of the WF image was sufficient to correlate fluorescence to individual dispersed viral particles, it was difficult to resolve individual viral particles within clusters (Fig. 3c). In contrast, the lateral resolution of the SR reconstruction allowed correlation of fluorescent signal to the cores of individual virus particles (Fig. 3d). The precision with which the YFP signal could be localised to virus particle structure in WF-EM overlays compared with SR-EM overlays was assessed using a standard 2-colour Pearson’s correlation coefficient measurement; a more negative coefficient reflecting a stronger correlation, since the viral particles were darker in the SEM image, and brightness was therefore anti-correlated. The WF-EM correlation was found to be −0.091, −0.002 and −0.053 (Fig. 3c, left to right, respectively), whereas the SR-EM correlation was considerably higher, at −0.165, −0.135, and -0.147 (O) (Fig. 3d, left to right, respectively), confirming the visual observation that in these images the SR signal was more strongly localised to viral particles.

### 2.4 GFP-C1 blinks *in vacuo*, enabling SR CLEM localisation of the DAG in cell membranes

**Fig. 4:**
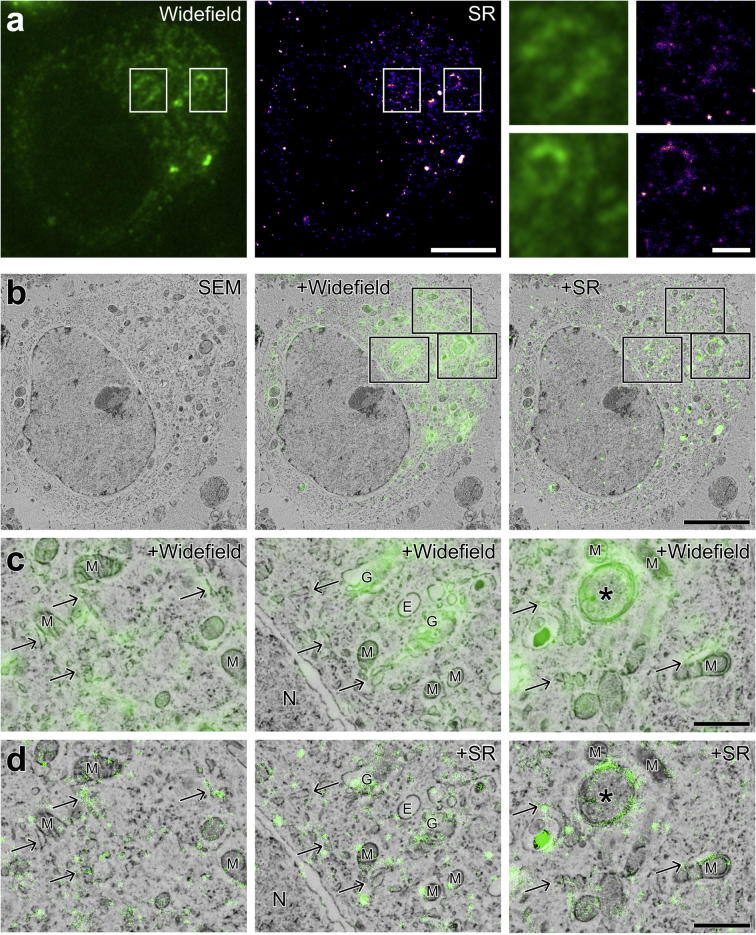
Comparison of WF to SR highlights significantly improved lateral resolution: GFP-C1. a: WF image showing localisation of the GFP-C1 construct (and hence the lipid DAG) within a HeLa cell, and SR reconstruction from a sequence of approximately 30,000 images, representing 71,413 individual localisations. Enlargements of the boxed areas show the improved precision of GFP-C1 localisation from SR based image reconstruction. b: Micrograph showing the BSE signal from an individual cell expressing the GFP-C1 construct with WF image and SR reconstruction overlaid. c, d: Enlargements of the boxed areas in B showing localisation of the GFP signal to membranes. The improved lateral resolution of the SR-EM overlay reveals DAG within Golgi stacks, endoplasmic reticulum (arrows), and a putative autophagosome (asterisk). G: Golgi, M: mitochondria, N: nucleus. Scale bars - 5 µm (a, b), 1 µm (A inset, c, d).

Since GFP has previously been shown to blink on a longer timescale than YFP (Bagshaw and Cherny, 2006, Dickson et al., 1997), the experiments were repeated to assess whether *in vacuo* SR imaging with GFP was feasible in sections of resin-embedded HeLa cells expressing the GFP-C1 construct. We found that GFP also blinks well in IRF sections *in vacuo* (SMovie 4). Reconstructed SR images of GFP-C1 had a resolution of 87.5 ± 27.7 nm, measured by assessing seven 2.7 μm^2^ patches using FRC (Fig. 4a); Composite images (Fig. 4b) were generated as before, showing an increase in the precision of correspondence between GFP signal and membrane structures in SR-EM overlays compared with WF-EM overlays. In the SR images, it was possible to localise DAG to membrane subdomains within the Golgi apparatus, cristae and perimeter membranes of mitochondria, endoplasmic reticulum and the perimeter membrane of a putative double-membrane autophagosome (Fig. 4c and d). These organelle localisations could not have been identified from fluorescence data alone, and required EM to add ground-truth to the SR reconstructions.

### 2.5 Vacuum pressure influences FP blinking

**Fig. 5:**
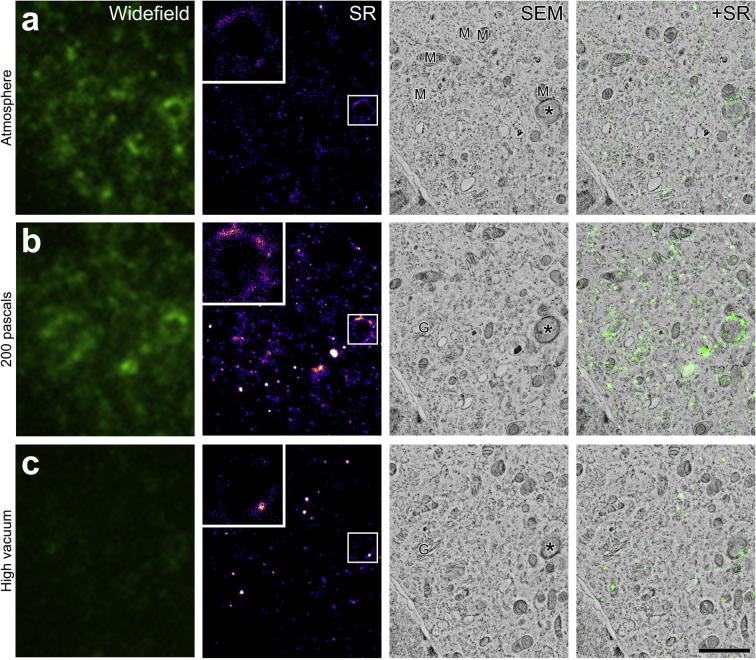
The quality of SR image reconstructions is directly influenced by the vacuum pressure at the time of acquisition. a: WF, SR and SEM images showing a cropped area of the GFP-C1 expressing cell shown in Fig. 4, acquired with the chamber at atmospheric pressure. The reconstruction represents ∼ 6,000 localisations. b: An area matching that shown in A from the preceding section in the series, acquired at a chamber pressure of 200 Pa, maintained at partial pressure using water vapour. The SR reconstruction represents ∼25,000 localisations. c: An area matching that shown in A and B from the next section in the series, acquired with the chamber pumped to high vacuum (10^−3^ Pa). The SR reconstruction represents ∼4,000 localisations. G: Golgi, M: mitochondria. Asterisk: putative autophagosome. Scale bar – 2 µm.

To assess the effect of vacuum pressure on FP blinking, we acquired SR images at atmospheric pressure and 10^−3^ Pa to complement the data gathered at 200 Pa (Fig. 5). Had we collected three sequential SR datasets from the same region at different vacuum pressures, photobleaching would influence the comparison between reconstructions. Therefore, we collected one dataset from each of three sequential 200 nm sections, in which some of the same organelles appeared (e.g. a putative autophagosome; Fig. 5). Surprisingly, there was a 76% reduction in the number of individual molecule localisations for a given region of interest at atmospheric pressure (∼6,000 localisations; Fig. 5a) compared with 200 Pa (∼25,000 localisations; Fig. 5b). At 10^−3^ Pa (Fig. 5C, high vacuum), the number of individual molecule localisations was reduced by 86% compared with 200 Pa (∼4,000 localisations at 10^−3^ Pa vs. ∼25,000 localisations at 200 Pa). In all vacuum conditions, the average number of localisations per frame showed a sharp decline over the initial 5,000 images (SFig. 1). This effect was most pronounced at atmospheric pressure where the number of localisations declined rapidly towards zero. At 200 Pa, the decline was less rapid and later stabilised, with localisations detected throughout the image sequence. At high vacuum, localisations were also detected throughout the image sequence, but with a lower average. Taken together, these findings suggest that, of the vacuum pressures tested, 200 Pa partial pressure of water vapour represents an optimal ‘sweet-spot’ for both fluorescence intensity (Brama et al., 2015) and blinking of GFP in IRF sections.

**Fig. 6:**
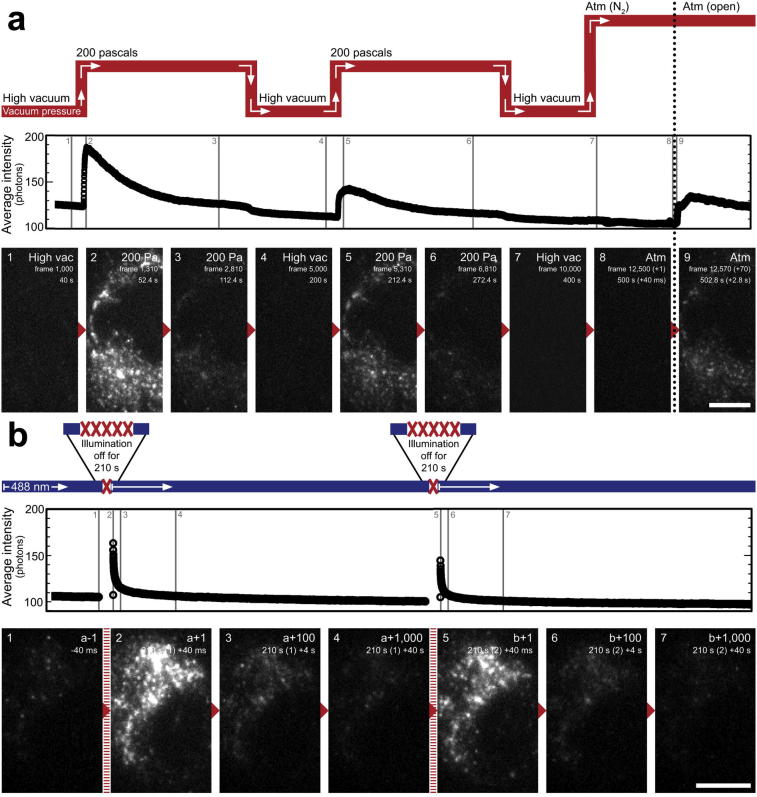
FP blinking is directly influenced by vacuum pressure and duration of illumination. A: Single frames and a graph of average intensity per frame taken from raw SR image acquisition sequences showing the effect of cycling chamber pressure, and illumination rest periods at 200 Pa, on FP blinking (see also supplementary movies 5 and 6). For each image, the approximate point at which it was recorded is indicated on the intensity graph. The chamber was cycled from 10^−3^ Pa → 200 Pa → 10^−3^ Pa → 200 Pa → 10^−3^ Pa → atmosphere. Frame number and time elapsed in seconds is shown on each image. At frame 1,000, the chamber pressure was set to 200 Pa; the fluorescence intensity and number of actively blinking molecules increased substantially for a short interval once the chamber reached 200 Pa (image 2). After 4,000 frames the chamber was returned to 10^−3^ Pa. After 5,000 frames, the chamber pressure was set to 200 Pa; the FP intensity and blinking again increased for a short interval once 200 Pa was reached (image 5). In both cases, the increase in intensity was short lived, and declined significantly over the next 2,000 frames to reach a similar pre-cycle level. The cycling process could be repeated multiple times, with similar results each time, though the first cycle was brightest at 200 Pa. The chamber was vented with dry nitrogen after 10,000 frames, which completed by 12,500 frames, and the chamber door was opened. After a short delay (frames recorded since opening the chamber door shown in brackets), FP intensity and blinking again increased, though to a lesser degree than when cycling from 10^−3^ Pa to 200 Pa. In B, the chamber pressure was maintained at 200 Pa and the light source was switched on/off at defined intervals to examine the effect of a ‘rest’ period on fluorophore response. Shown here are single frames from a sequence of ∼15,000 images; at frame ‘a’, the light source was turned back on after a 5,000 image break (∼3.5 mins), and again at frame ‘b’ after a further 5,000 image break (∼3.5 mins). In both cases, the intensity of fluorescence and number of blinking FPs increased temporarily, and declined over the following 1,000 frames (indicated top right of images). Scale bars – 5 µm.

We then showed that it was possible to modulate the blinking of GFP by cycling through different vacuum pressures sequentially whilst maintaining continuous illumination (Fig. 6, SMovie 5). After establishing high vacuum, the chamber could be repeatedly cycled from 10^−3^ Pa to 200 Pa and back again, each cycle taking just over 3 min to complete (Fig. 6a). SR image acquisition began when the laser power was increased to a power density of 330 W/cm^2^ in high vacuum, and images were acquired with 40 ms exposures. Over each cycle, GFP blinking was suppressed at 10^−3^ Pa (Fig. 6a, images 1 and 4), and then recovered at 200 Pa (Fig. 6a, images 2 and 5). Interestingly, though GFP blinking reduced over time at 200 Pa (Fig. 6a, images 3 and 6), cycling the vacuum to 10^−3^ Pa and then back to 200 Pa enhanced blinking compared with that expected had the chamber been maintained at 200 Pa with continuous illumination for an equivalent time period (Fig. 6a, image 5). Curiously, interrupting continuous illumination had a similar but less robust effect on blinking (Fig. 6b; SMovie 6). Here, a defined period with the illumination switched off (∼3.5 min) resulted in a short-lived recovery of blinking (Fig. 6b, image 2), which then reduced again over the next 30–40 s. Subsequent additional short periods of ‘rest’ also generated reproducible short-lived restorations of blinking (Fig. 6b, image 5).

During the course of these cycling experiments, there was a noticeable delay in the recovery of GFP blinking when moving between vacuum pressures. When cycling from 10^−3^ Pa to 200 Pa, this delay was very short and synchronised with the introduction of water vapour into the SEM chamber. When moving from 10^−3^ Pa to atmospheric pressure (relative humidity of around 70%), GFP blinking recovered quickly once the chamber door was opened, after a delay of some 2–3 s. This may be attributable to the time taken to rehydrate the fluorophores by diffusion of water into the chamber and section, adding to the evidence that water is essential for GFP blinking. On venting the chamber with dry nitrogen gas, GFP blinking was suppressed, in agreement with our previous observation that GFP intensity drops when dry nitrogen is used to create partial pressure instead of water vapour (Brama et al., 2015) (Fig. 6a, image 8).

We next investigated how electron contrast affected GFP blinking. We previously reported that IRF sample preparation resulted in a reproducible gradient of electron contrast, from heavy membrane-staining at the edge of the cell pellet exposed directly to the uranyl acetate stain, to lighter membrane-staining at the opposite side. This was associated with an opposing gradient of fluorescence intensity across the cell layer (Peddie et al., 2014). We now report a similar gradient in FP blinking that correlated positively with fluorescence intensity and negatively with electron contrast. Cells of interest expressing the GFP-C1 construct were chosen from an area considered to represent a good balance between electron contrast and fluorescence intensity (‘balanced contrast’; SFig. 2a and b), and from an area considered to be optimal for electron contrast, but sub-optimal for fluorescence intensity (‘high contrast’; SFig. 2c and d). As might be expected, given that fluorescence intensity diminishes substantially in the presence of high concentrations of heavy metal contrast agents, SR reconstructions from high contrast cells contained fewer individual localisations in comparison to those from balanced contrast cells (∼51,000 localisations for ‘high contrast’ vs.∼168,000 localisations for ‘balanced contrast’; SFig. 1), though ultrastructure was superior in high contrast cells.

Finally, we imaged dry-mounted 200 nm IRF sections containing YFP-A3 vaccinia using an N-STORM microscope system, to compare the performance of a commercial SR microscope to the SECOM platform (SFig. 3, SMovies 7, 8 and 9). We found that the frequency of FP blinking was similar during the initial phase of data collection (SFig. 3 frames 1 and 50, SMovies 7, 8 and 9). However, FP blinking frequency reduced more quickly in the N-STORM dataset when compared to the SECOM at 200 Pa (SFig. 3 frames 200 and 2,000, SMovies 7 and 9). This is illustrated by maximum intensity projections from early and late points in the data collection (frames 300–500 vs. frames 2,100–2,300). The signal to noise ratio also worsened in the later stages of data collection at atmospheric pressure, further highlighting a reduction in FP blinking (SFig. 3 frame 2,000, SMovies 7, 8 and 9). In total, the SECOM dataset recorded at 200 Pa contained ∼2.27× more localisations than were detected using N-STORM. The results at atmospheric pressure were more comparable, with ∼1.46× more localisations detected in the SECOM dataset. Although the absolute number of individual molecule localisations cannot be directly compared because of differences in the sections imaged and the microscope systems, these findings provide further evidence that FP blinking in IRF sections can be driven more effectively at 200 Pa.

## 3 Discussion

One of the major challenges in correlative microscopy is to achieve a level of localisation precision of biomolecules to cellular structures that rivals more traditional on-section immunogold labelling methods. We addressed this challenge by taking advantage of a novel blinking property of FPs in cells embedded in an acrylic resin, combined with an imaging workflow for single molecule localisation microscopy *in vacuo* in an integrated light and SEM, and achieved super-resolution molecular localisations of ∼85 nm in the lateral plane. Axial resolution was determined by physical section thickness, and precise overlay of images from SR and EM modalities was possible because the sample did not move between light and electron imaging.

This workflow delivers several advances in super-resolution imaging. The overlaid SR-EM images provide a new ‘ground truth’ method for testing super-resolution reconstruction algorithms and parameters. For example, we determined that closely opposed virus particles sometimes appear as elongated, curved structures rather than punctate point sources in SR images, information that can be used to improve reconstruction software to remove sample-specific errors. Furthermore, we propose that serial IRF sections could be imaged using a standard fluorescence microscope equipped with a vacuum stage (as commonly used in materials research and available off-the-shelf) and lasers, enabling multi-colour 3-dimensional SR imaging using standard FPs without the need for multiple buffers tailored to each fluorophore. In this case, sample preparation would be simplified as electron contrast would be unnecessary, and the blinking activity of FPs would likely be improved in the absence of metal staining. Automation of this process could lead to the generation of 3D SR molecular localisations through large volumes of cells and tissues.

The workflow also delivers advances in correlative microscopy. In post-embedding CLEM experiments on thin sections, it is necessary to treat the section for each imaging modality. In practice, this usually means wet mounting of the sections for light microscopy, followed by rinsing and additional contrasting for electron microscopy. Each of these steps can potentially alter the specimen, causing section distortion or shrinkage, or subtle changes in molecular composition. The physical transfer step between imaging modalities also makes the correlation of data significantly more challenging, particularly in cases where the relationship between protein location and structure is unknown. Collecting sections directly on a rigid conductive coverslip and imaging dry within the partial vacuum of the ILSEM means that it is possible to avoid these complicating factors. The high precision with which SR and EM images can be correlated when using the ILSEM is therefore a major strength of our approach. It also eliminates many of the complexities of pre-embedding CLEM image alignment. Furthermore, the workflow has great potential for multi-colour molecular localisation through large cell and tissue volumes, by acquiring sequential SR images across serial sections via ‘integrated super-resolution array tomography’.

This study also revealed some unexpected properties of resin-embedded FPs. First, YFP and GFP display strong stable blinking *in vacuo* when embedded in resin after freeze substitution with a small percentage of water. Second, YFP and GFP blink better in resin at 200 Pa partial pressure of water vapour than they do at either atmospheric pressure, or at 10^−3^ Pa. This was unexpected because the intensity of YFP and GFP in widefield imaging mode was higher at atmospheric pressure than at 200 Pa (Brama et al., 2015). We postulate that this 200 Pa ‘sweet-spot’ benefits from removal of damaging reactive oxygen species (ROS) from the fluorophores by the vacuum, whilst the water vapour within the SEM chamber maintains the hydration state of the fluorophores. It is also possible that the conditions in the ILSEM chamber allow a subpopulation of FP molecules to blink stably and repeatedly over time, and/or additional populations of FP molecules to fluoresce by revealing intact molecules that were previously in a non-emitting state, and/or drive more effective switching between emitting and long-lived dark states. Finally, it was possible to control the blinking of FPs over time by modulating the vacuum conditions under continuous illumination, or switching the illumination off for a rest period. These findings suggest that 488 nm illumination drives many of the fluorophores into a long-lived dark state, rather than causing irreversible bleaching.

The reversal of photobleaching in GFP and its variants has been described previously, with possible mechanisms including altered equilibrium between protonated and deprotonated states (Brejc et al., 1997, Schwille et al., 2000, Sinnecker et al., 2005), and/or structural isomerisation (Gayda et al., 2012, Henderson et al., 2007, Niwa et al., 1996). Given the response seen here in resin sections, it is likely that the same phenomenon persists in resin embedded specimens. The observation that the duration of recovery was enhanced during vacuum cycling in comparison to illumination cycling suggests that more than one mechanism could be involved in recovery, and points toward the existence of multiple distinct dark states. Alternatively, the conditions experienced by fluorophores during vacuum cycling may be responsible for driving a more efficient change in the equilibrium of light and dark states. A clear relationship between pH and the fluorescence dynamics of GFP derivatives has also been demonstrated (Hanson et al., 2002, Haupts et al., 1998, Henderson et al., 2007, Schwille et al., 2000, Sinnecker et al., 2005), and warrants further examination with respect to the findings presented here. In future work, we will also investigate whether the blinking observed during vacuum or illumination cycling originated from the same FPs, or were ‘newly revealed’ FPs. If the latter were true, it would be advantageous to operate a cyclical imaging regime, thereby maximising the number of individual FPs from which localisations could be recorded, and potentially increasing resolution.

## 4 Materials and methods

### 4.1 Cell culture, constructs and transfection

Human cervical cancer epithelial HeLa cells were obtained from the American Type Culture Collection (ATCC, CCL-2). Cells were maintained in DMEM supplemented with 10% fetal bovine serum in 10 or 15 cm tissue culture dishes. EGFP-PKCε was made as previously described (Domart et al., 2012). Briefly, cells were transfected with 0.5 μg DNA (for 3.5 cm MatTek dishes) of each construct using Lipofectamine LTX and PLUS reagent (Invitrogen, Life Technologies Ltd, Paisley) in OPTIMEM medium (Gibco, Life Technologies Ltd, Paisley). The transfection mix was added to the cells in antibiotic-free medium. Microscopy and/or fixation was performed 18–24 h after transfection. HeLa cells were also infected with Vaccinia virus expressing YFP-A3 (Arakawa et al., 2007) at a multiplicity of infection of 2 in serum free DMEM. Infected cells were fixed at 18 h post infection for downstream processing.

### 4.2 In-Resin fluorescence (IRF) preparation

HeLa cells infected with Vaccinia virus were fixed in 4% formaldehyde in 0.1 M phosphate buffer to deactivate the virus prior to further processing. IRF preparation was performed as described previously (Peddie et al., 2014). Briefly, cells were spun in a 1.5 ml Eppendorf tube to form a pellet, resuspended in an equal volume of media containing 10% BSA, and maintained at 37 °C. 50 μl of the cell suspension was pelleted in a blocked 200 μl pipette tip, from which the cells were transferred to membrane carriers after removal of the blockage and loaded into the EMPACT2 high pressure freezer using the rapid transfer system (Leica Microsystems, Vienna). Carriers containing frozen cells were stored under liquid nitrogen.

Quick freeze substitution (QFS) was performed using a modified version of the method described by McDonald and Webb (2011). The substitution medium was 5% H_2_O in acetone, with 0.1% uranyl acetate diluted from a 20% stock in methanol. After freeze substitution, the cells were infiltrated with HM20 resin at −50 °C in an automated freeze substitution unit (AFS2; Leica Microsystems, Vienna) and polymerised under 360 nm UV light over 48 h, before warming to room temperature.

Polymerised blocks were trimmed and stored at room temperature in the dark. The membrane carriers were carefully trimmed away by hand, and the blocks cut and trimmed perpendicular to the cell layer to allow examination of the full depth of the layer. 200 nm sections were cut using a UC7 ultramicrotome (Leica Microsystems, Vienna), collected on indium tin oxide (ITO) coated glass coverslips (Delmic B.V., Delft), and the coverslip attached to a specimen holder.

Fixed mounted whole cells containing either GFP-C1 or YFP-A3 were cultured directly on glass coverslips, fixed as described above, and mounted using Citifluor AF4.

### 4.3 Integrated light and SEM (ILSEM)

ILSEM was performed on the same day as sectioning using a SECOM light microscope platform (Delmic B.V., Delft) with Nikon Plan Apo 100×/1.4 objective and vacuum compatible immersion oil (Delmic B.V., Delft), mounted on a Quanta 250 FEG SEM (FEI Company, Eindhoven). A schematic illustrating the overall imaging workflow adopted for sequential fluorescence, super-resolution, and SEM imaging is shown in Fig. 2.

Diffraction-limited imaging was performed as previously described (Peddie et al., 2014). Briefly, YFP or GFP fluorescence was stimulated by excitation with a 488 nm laser light source and multi-band filters (Di01-R405/488/594 dichroic, FF01-446/532/646-25 emission; Semrock, Rochester NY). Individual fluorescence images were collected using an EMCCD camera (iXon 897 Ultra; Andor Technology, Belfast) with exposure time of 2 s, and power density of 0.5 W/cm^2^ at the sample level. During all fluorescence imaging phases, the chamber was maintained at a partial pressure of 200 Pa, created using water vapour.

For super-resolution localisation-based imaging of specimens containing multiple fluorophores using high laser powers, it was necessary to further filter emission wavelengths using an individual bandpass filter (FF01-520/35-25; Semrock, Rochester, NY), which was placed directly in front of the camera. YFP or GFP fluorescence was again stimulated by excitation with a 488 nm laser light source at a power density of 330 W/cm^2^. Control of lasers and EMCCD camera were transferred to Micromanager (Edelstein et al., 2014) and the following camera parameters were set: −85 °C temperature set point, photon count convert, frame transfer on, EMCCD gain 200, preamplifier gain +3, readout speed 17 MHz, vertical clock speed +1.70. Sequences of ∼30,000 images were collected, each with an exposure time of 40 ms. The image sequences were processed for analysis as detailed below using ThunderSTORM (Ovesny et al., 2014).

For SEM imaging, the system was pumped to high vacuum (∼10^−3^ Pa). The vCD backscatter detector (FEI Company, Eindhoven) was used at a working distance of 7.3 mm, and inverted contrast images were acquired (2.5 keV, spot size 3.5, 30 μm aperture, and pixel dwell time of 60 μs for a 3,072 * 1,536 image frame).

### 4.4 Data analysis

The theoretical resolution of the SECOM platform using a 488 nm light source and 100×/1.4 objective lens was calculated as 213 nm. The full width half-maximum (FWHM) of the system was measured using 30 nm fluorescent beads at ∼265 nm. The calculated pixel size of the iXon EMCCD camera was 107.8 nm.

Several freely available reconstruction packages were tested during preliminary super-resolution data acquisition in order to determine which worked better with the data generated by the ILSEM-based system, including 3B (Cox et al., 2012), CSSTORM (Zhu et al., 2012), Localizer (Dedecker et al., 2012), Octane (Niu and Yu, 2008), QuickPALM (Henriques et al., 2010), RainSTORM (Eric et al., 2013) and RapidSTORM (Wolter et al., 2012). The patterns of particle localisation obtained were remarkably similar, but with tremendous variation in processing time and flexibility of operation from a user standpoint (refer also to Sage et al. (2015) for a review). The Fiji plugin ThunderSTORM was used to reconstruct all data from the imaging sequences shown here (Ovesny et al., 2014, Schindelin et al., 2012). ThunderSTORM represented an acceptable balance between overall processing time and perceived quality of output when compared to the electron imaging ‘ground truth’ data; it also offered more flexibility for interactive data post-processing.

Camera setup was performed according to the EMCCD specification and EM gain applied during acquisition. Given the calculated system resolution, a fitting radius of 4 pixels was used, with sigma of 1.6 pixels, and multi-emitter fitting analysis. Post-processing of the detected molecule localisations was carried out in the following sequence: remove duplicates (uncertainty_xy); filter (intensity > 5 & intensity < 2,000 & frame > 500); density filter (radius 100 nm, neighbours 5); drift correct (cross correlation, bins 5, magnification 5.0); merging (xy distance 20, off frames 1).

Reconstructions were generated using averaged shifted histograms at a magnification of 10× (for low magnification overlays; histogram mapped to a range of 0–30) or 20× (for high magnification overlays; histogram mapped to a range of 0–14.74) to avoid interpolation when overlaying onto electron micrographs. A ‘Fire’ LUT was applied before exporting the images as 16 bit RGB TIFFs for overlay onto the corresponding electron micrographs.

Electron micrographs were adjusted to reduce image noise and enhance contrast, and composite images of dual signals were generated as described previously (Peddie et al., 2014). Movies of the image sequences were generated using iMovie (Apple). As the original image sequences were recorded with 16 bit greyscale depth, movies 3, 4, 7–9 were processed in Fiji (contrast normalisation; 0.1% saturated pixels) for on-screen presentation.

### 4.5 Imaging IRF sections with N-STORM

IRF sections of 200 nm thickness containing YFP-A3 vaccinia were collected as before, with the coverslip inverted and dry mounted on a standard microscope slide for compatibility with the N-STORM microscope stage. SR reconstructions were generated as above for each dataset with appropriate parameters for each microscope system; each of the datasets used for comparison in SFig. 3 was post-processed using matching parameters as detailed above.

## 5 Author contributions

CJP and LMC designed the experiments. MCD and XS produced the cell lines and fixed the samples. CJP processed the samples, and performed the imaging and super-resolution reconstructions. SC analysed the data for Pearson’s, FRC, and average localisations, and collected the N-STORM data. CJP and LMC wrote the manuscript with help from the other authors.

## Competing financial interests

The authors declare no competing financial interests.

## Materials and correspondence

Requests for materials and correspondence should be directed to CJP or LMC.

## Figures and Tables

**Supplementary Fig. 1:**
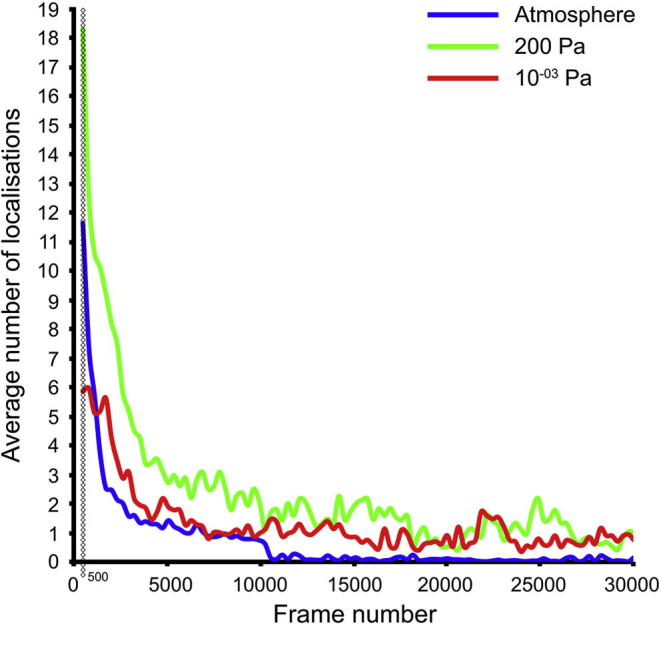
Comparison of the average number of localisations per frame at different chamber pressures. Graph showing the average number of localisations per frame at atmosphere, 200 Pa, and 10^−3^ Pa across each 30,000 image sequence from consecutive serial sections (200 Pa data shown in Fig. 4; cropped areas shown in Fig. 5). The first 500 frames were excluded from analysis as the EMCCD was close to saturation and FP blinking was difficult to observe (indicated by vertical line).

**Supplementary Fig. 2:**
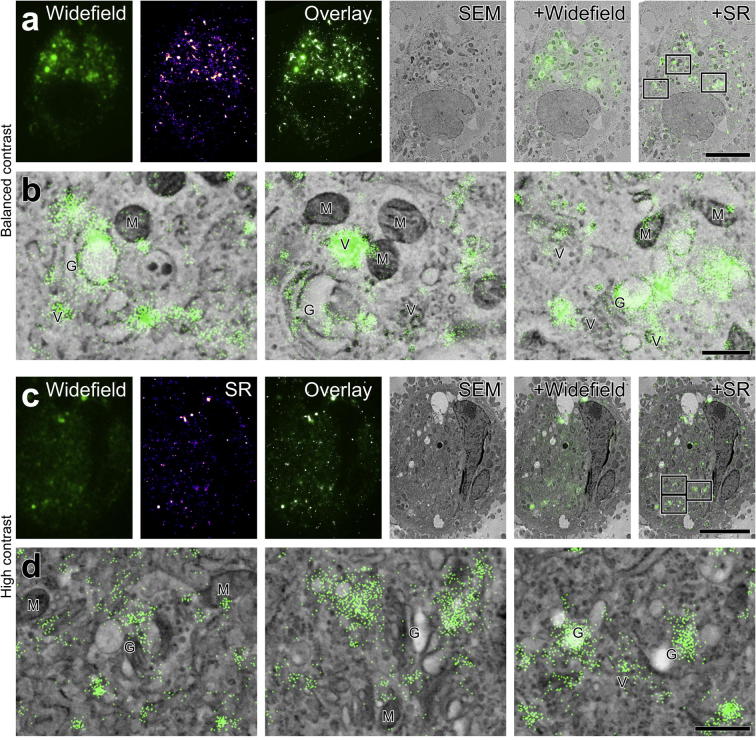
The quality of SR image reconstructions, and the number of individual molecule localisations detected, is inversely related to electron contrast. a: WF, SR reconstruction representing 167,796 individual localisations, and overlaid images from a ‘balanced contrast’ cell expressing the GFP-C1 construct, a similar contrast level to that shown in Fig. 3. Electron micrographs with overlaid WF and SR images are also shown. b: Enlargements of the boxed areas in A showing overlay of the SR reconstruction to specific membranous structures with a much higher degree of precision compared to the WF image alone. Cc: WF, SR reconstruction representing 51,494 individual localisations, and overlaid images from a ‘high contrast’ cell expressing the GFP-C1 construct. The signal to noise ratio in the WF image worsens as electron contrast increases, as does the number of localisations for SR reconstruction. Electron micrographs with overlaid WF and SR images are also shown. D: Enlargements of the boxed areas in c. As before, localisation of signal to specific membranous structures is possible, though it is clear that the decreased number of localisations reduces the certainty with which signals can be assigned to specific structures. G: Golgi, M: mitochondria, V: vesicles. Scale bars – 5 µm (a, c), 500 nm (b, d).

**Supplementary Fig. 3:**
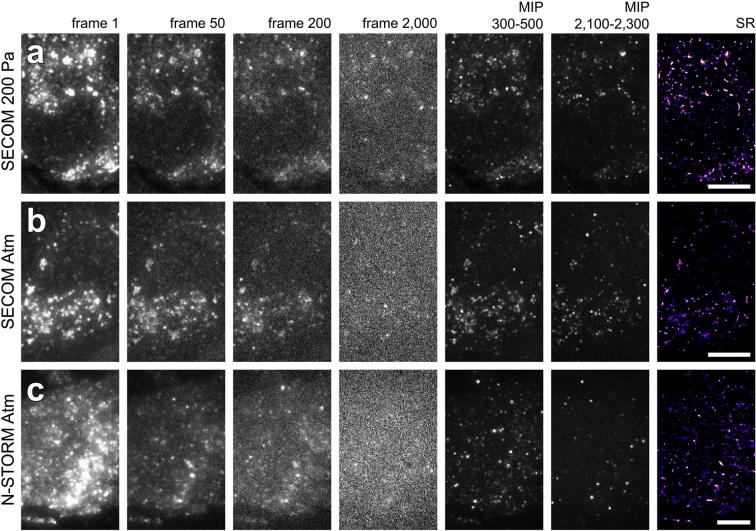
FP blinking persists at a higher frequency during imaging at 200 Pa using the SECOM platform (a) when compared to atmospheric pressure in the SECOM platform (b) and the Nikon N-STORM microscope (c). Single frames from a SR acquisition sequence acquired from cells containing YFP-A3 vaccinia in a dry mounted 200 nm thick IRF section. Each dataset was cropped to contain a single cell profile and comprises 2300 images (presented also in supplementary movies 7–9), illustrating the intensity of fluorescent signal at frame 1, 50, 200, and 2000. Note that, for clearer visualisation, contrast normalisation was applied to the 16 bit image sequences (0.1% saturated pixels). Maximum intensity projections from the raw data, and SR reconstructions (generated for each dataset using matching parameters), showed that the signal to noise ratio and number of localisations degraded more quickly in the N-STORM dataset (120,235 localisations were detected for the SECOM reconstruction in a, 77,344 for the SECOM reconstruction in b, and 52,904 for the N-STORM reconstruction in c). Scale bars – 5 µm.

## Appendix A Supplementary data

Supplementary movie 1 YFP blinking in fixed HeLa cells at atmospheric pressure.

Supplementary movie 2 GFP blinking in fixed HeLa cells at atmospheric pressure.

Supplementary movie 3 YFP blinking in IRF sections at 200 pascals.

Supplementary movie 4 GFP blinking in IRF sections at 200 pascals.

Supplementary movie 5 GFP blinking response in IRF sections to changes in vacuum pressure.

Supplementary movie 6 GFP blinking response in IRF sections to changes in illumination.

Supplementary movie 7 YFP blinking in an IRF section at 200 pascals acquired using the SECOM platform (single cell cropped from SMovie 3).

Supplementary movie 8 YFP blinking in an IRF section at atmospheric pressure acquired using the SECOM platform.

Supplementary movie 9 YFP blinking in an IRF section at atmospheric pressure acquired using a Nikon N-STORM microscope system.
