# Supplementary figures and images for: Correlative super-resolution fluorescence and electron microscopy using conventional fluorescent proteins in vacuo
Source: J Struct Biol. 2017 Aug;199(2):120–31. doi: 10.1016/j.jsb.2017.05.013 (PMC5531056; doi:10.1016/j.jsb.2017.05.013)

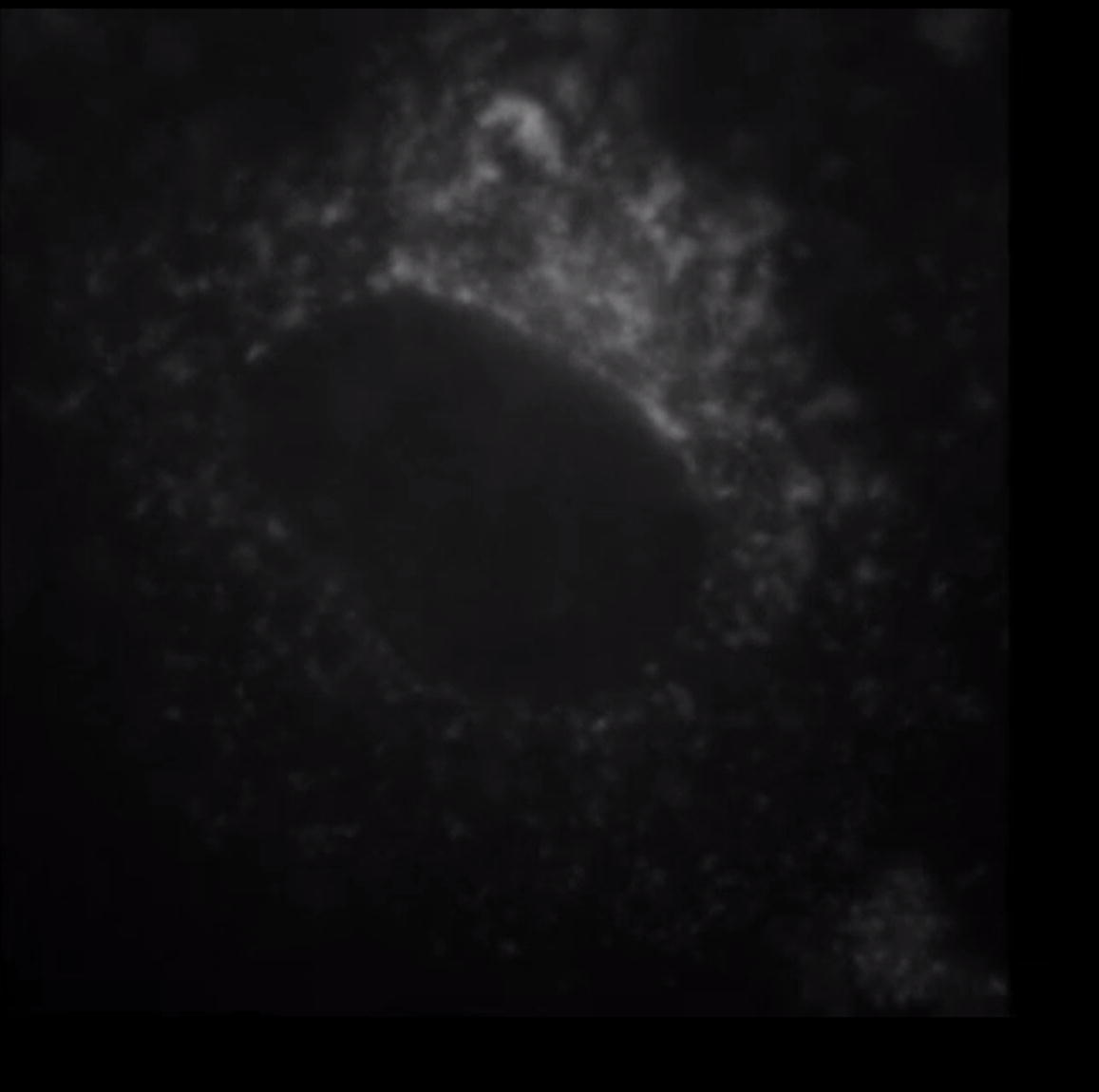

Supplement: Supplementary movie 1 — YFP blinking in fixed HeLa cells at atmospheric pressure. [file mmc1.jpg]

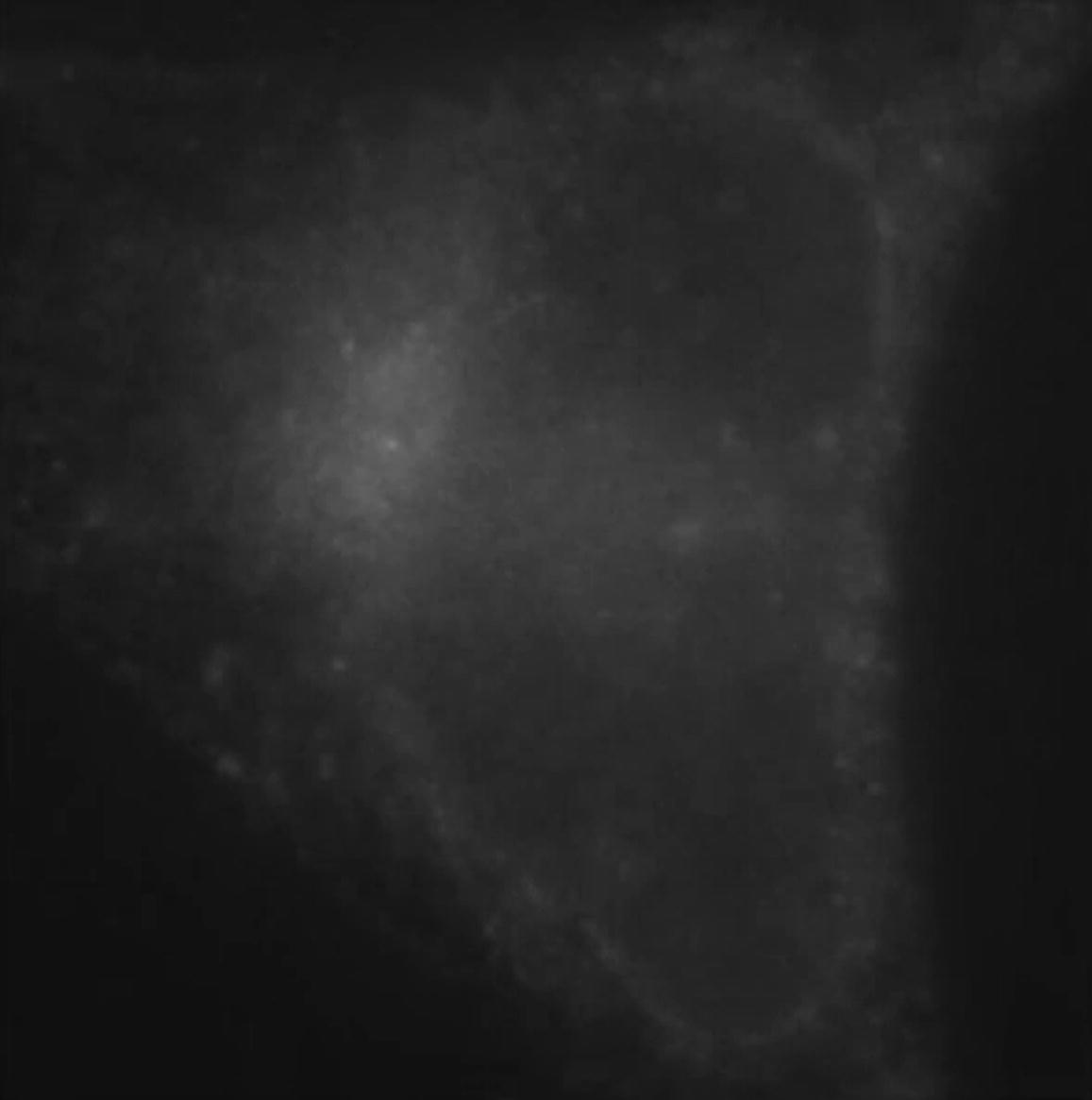

Supplement: Supplementary movie 2 — GFP blinking in fixed HeLa cells at atmospheric pressure. [file mmc2.jpg]

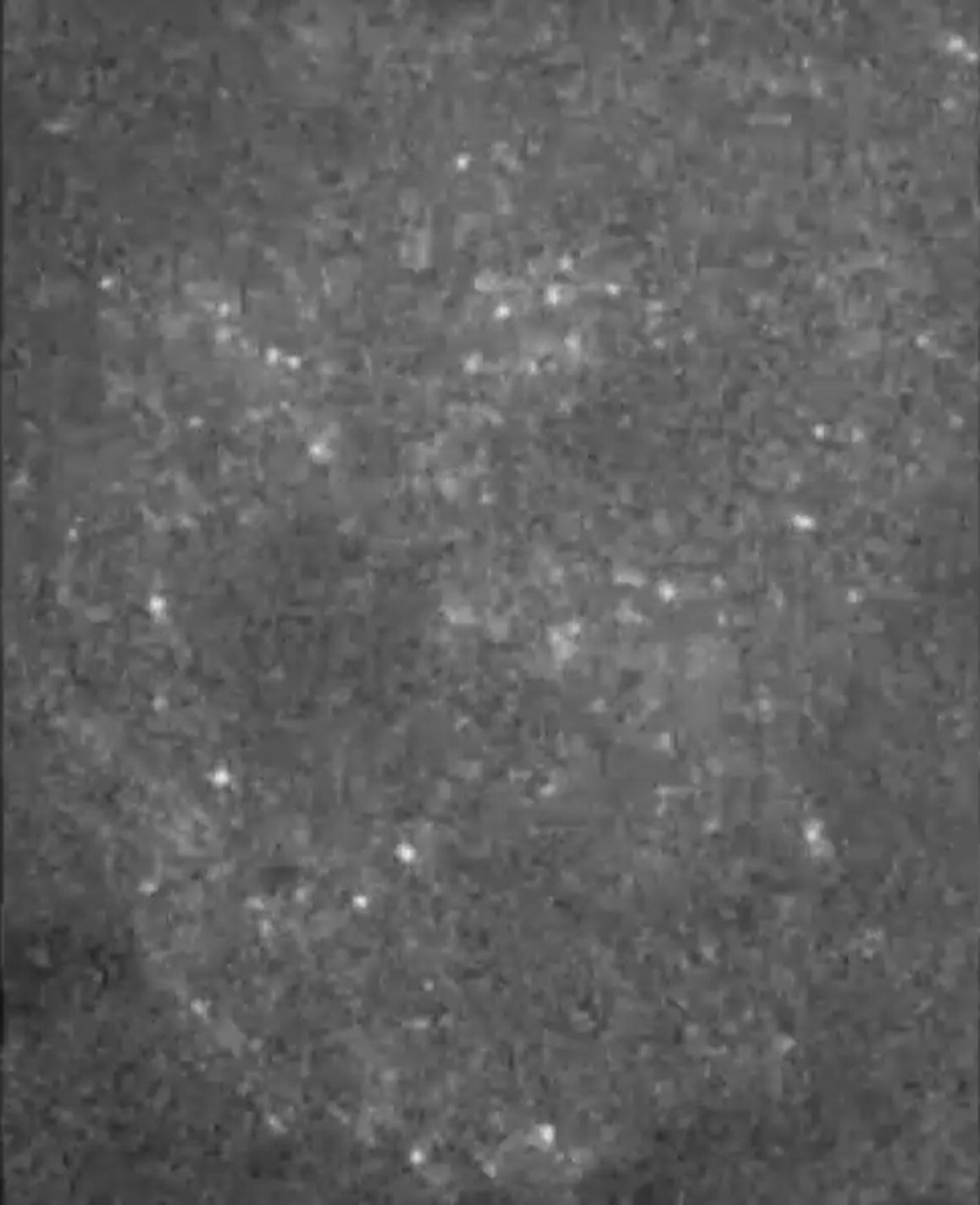

Supplement: Supplementary movie 3 — YFP blinking in IRF sections at 200 pascals. [file mmc3.jpg]

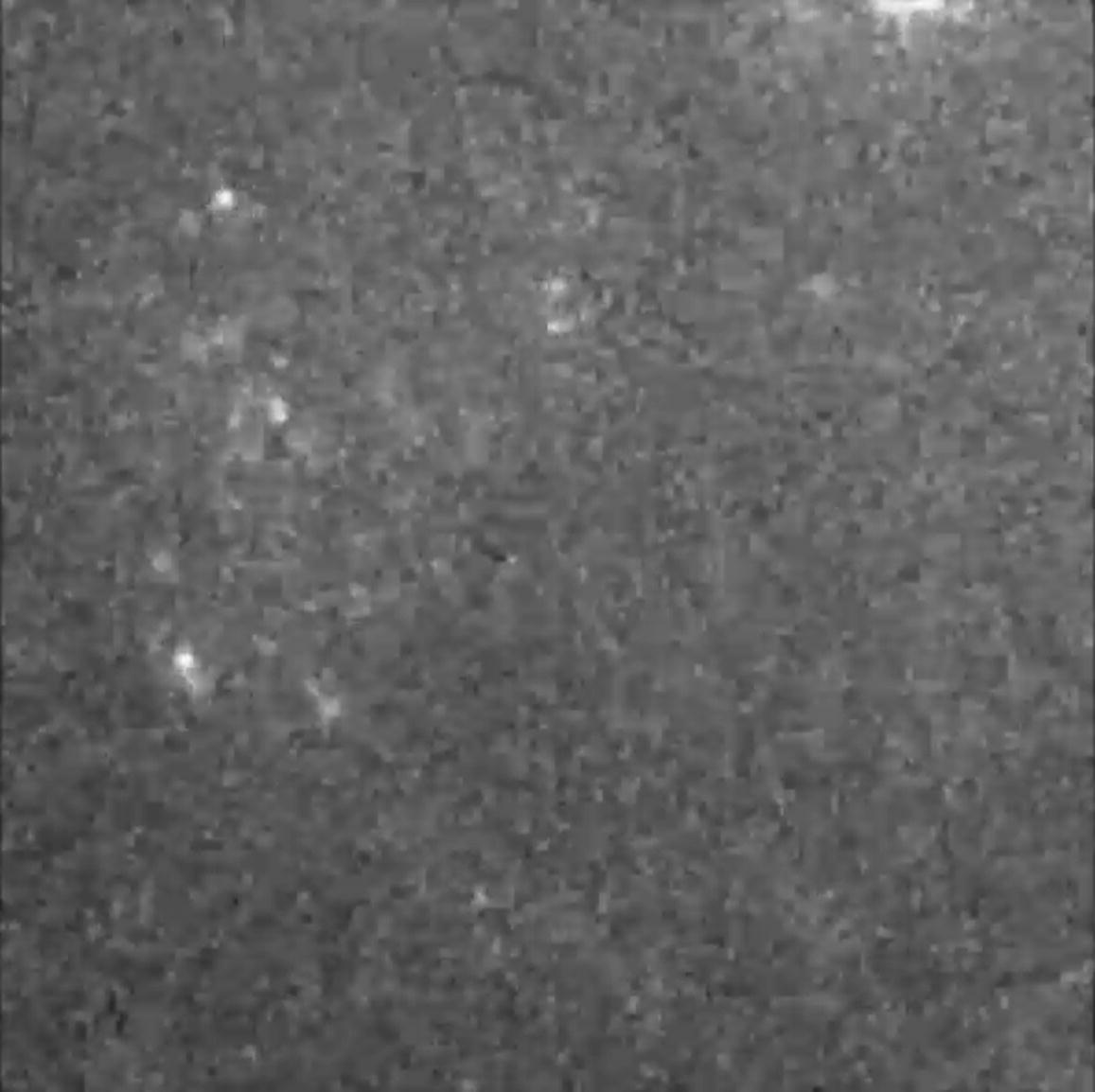

Supplement: Supplementary movie 4 — GFP blinking in IRF sections at 200 pascals. [file mmc4.jpg]

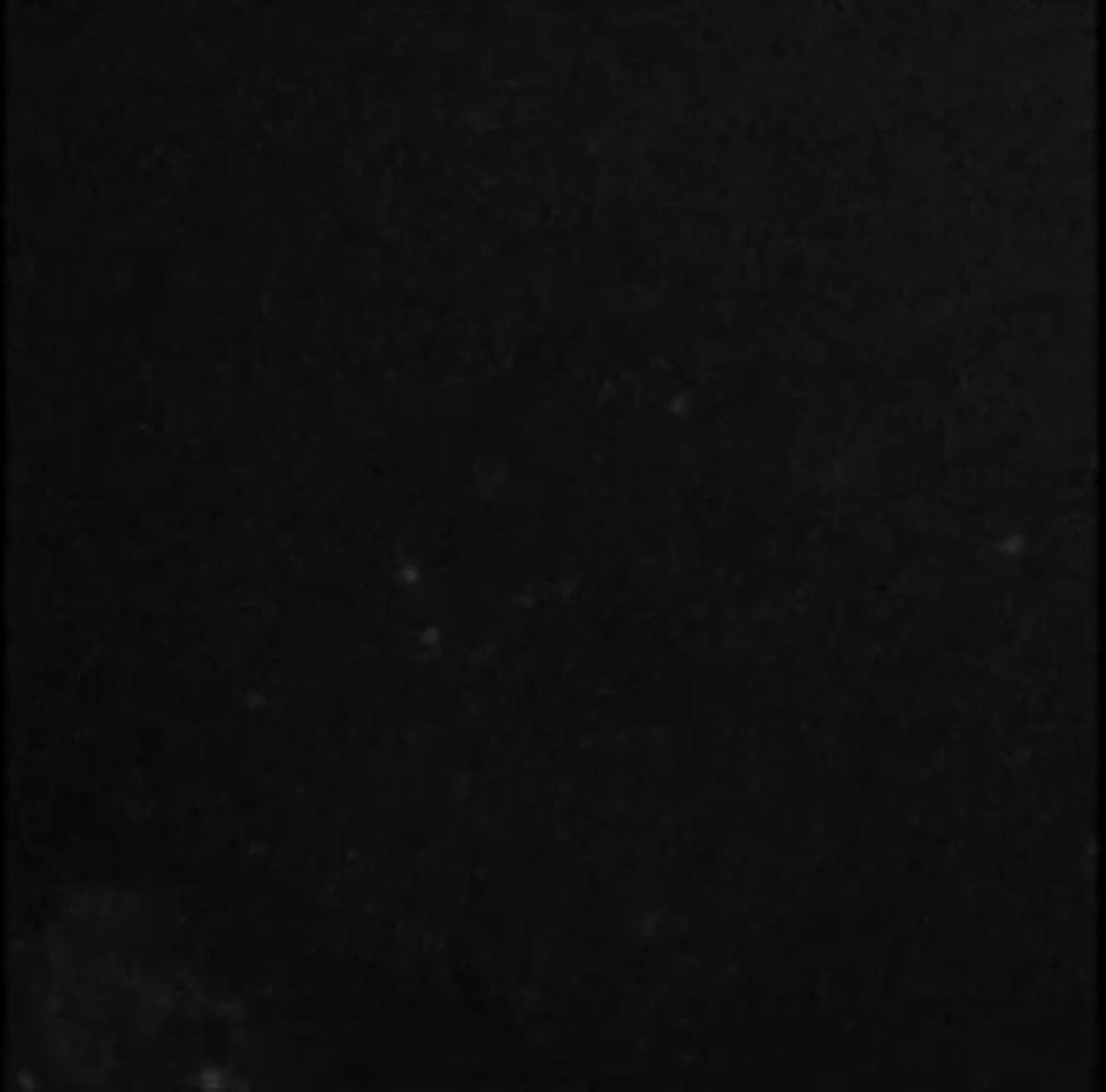

Supplement: Supplementary movie 5 — GFP blinking response in IRF sections to changes in vacuum pressure. [file mmc5.jpg]

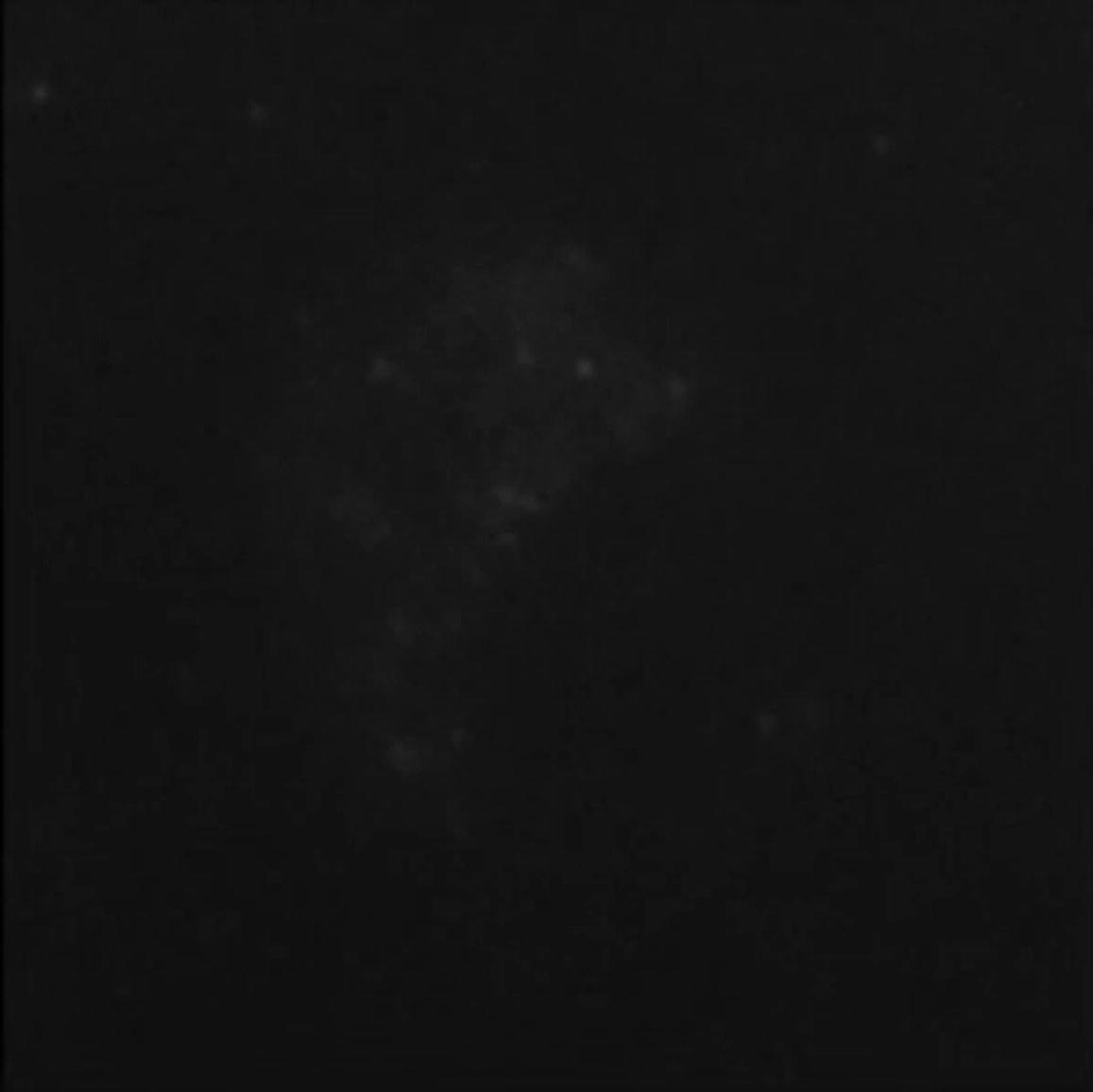

Supplement: Supplementary movie 6 — GFP blinking response in IRF sections to changes in illumination. [file mmc6.jpg]

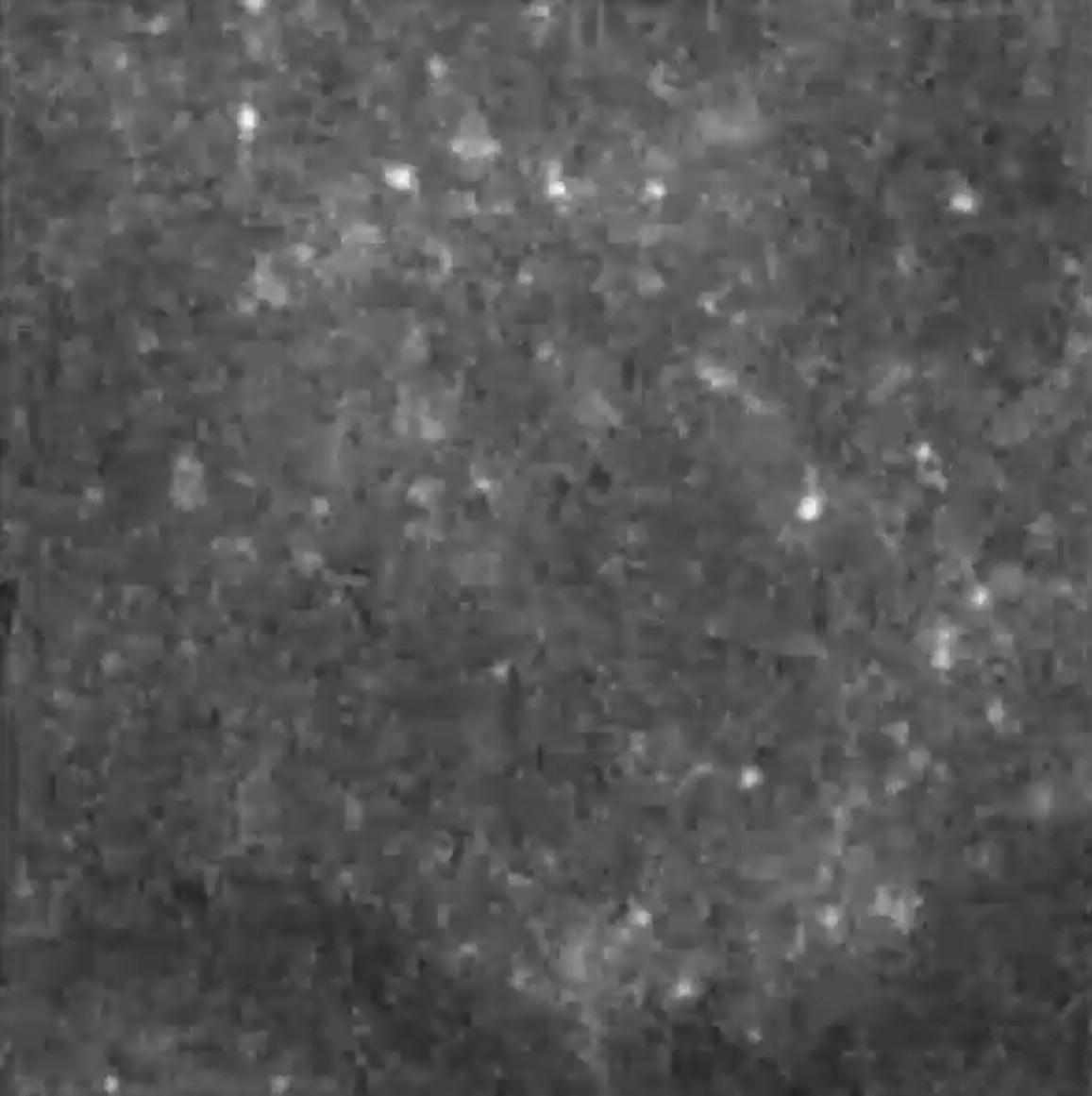

Supplement: Supplementary movie 7 — YFP blinking in an IRF section at 200 pascals acquired using the SECOM platform (single cell cropped from SMovie 3). [file mmc7.jpg]

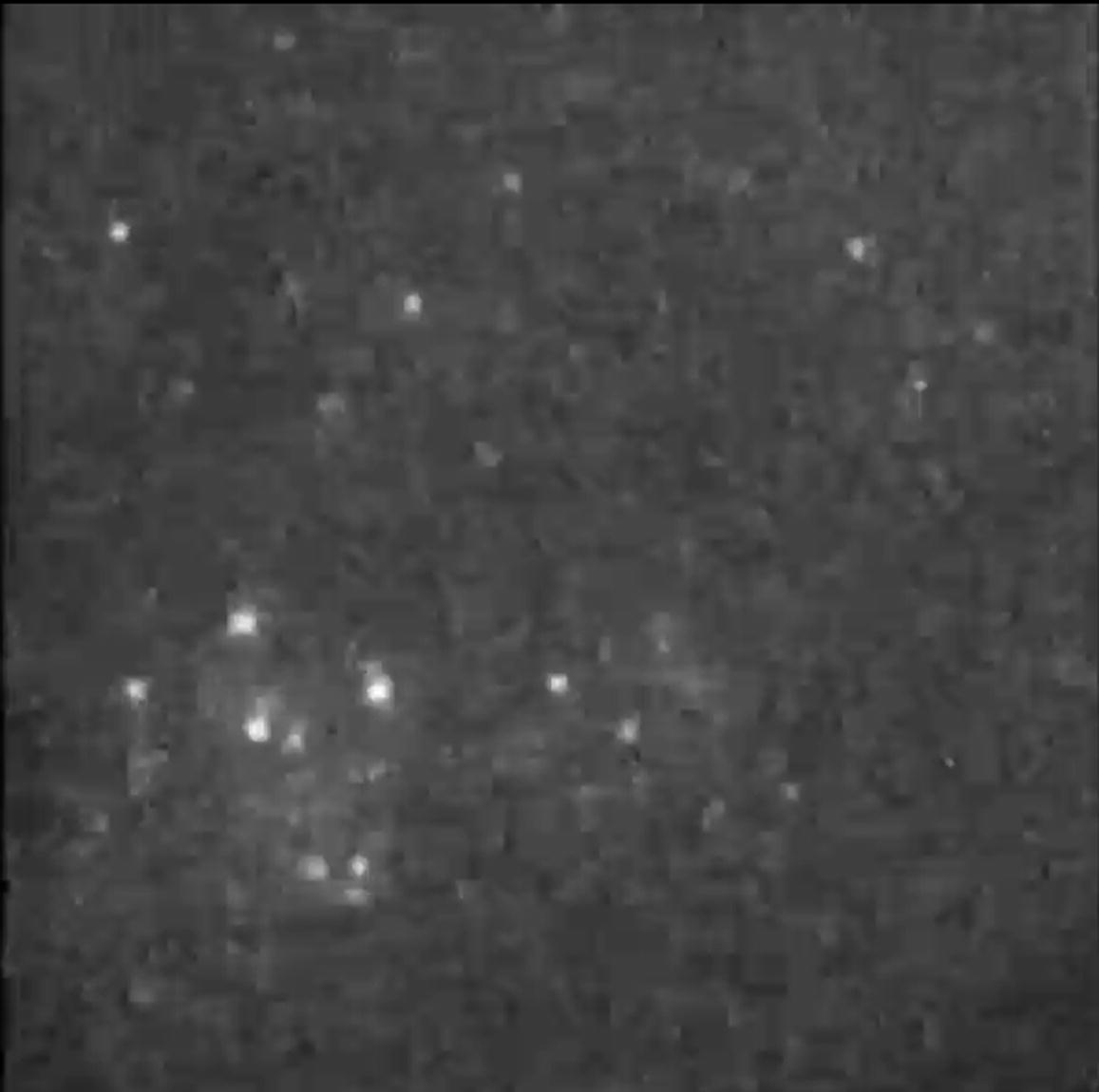

Supplement: Supplementary movie 8 — YFP blinking in an IRF section at atmospheric pressure acquired using the SECOM platform. [file mmc8.jpg]

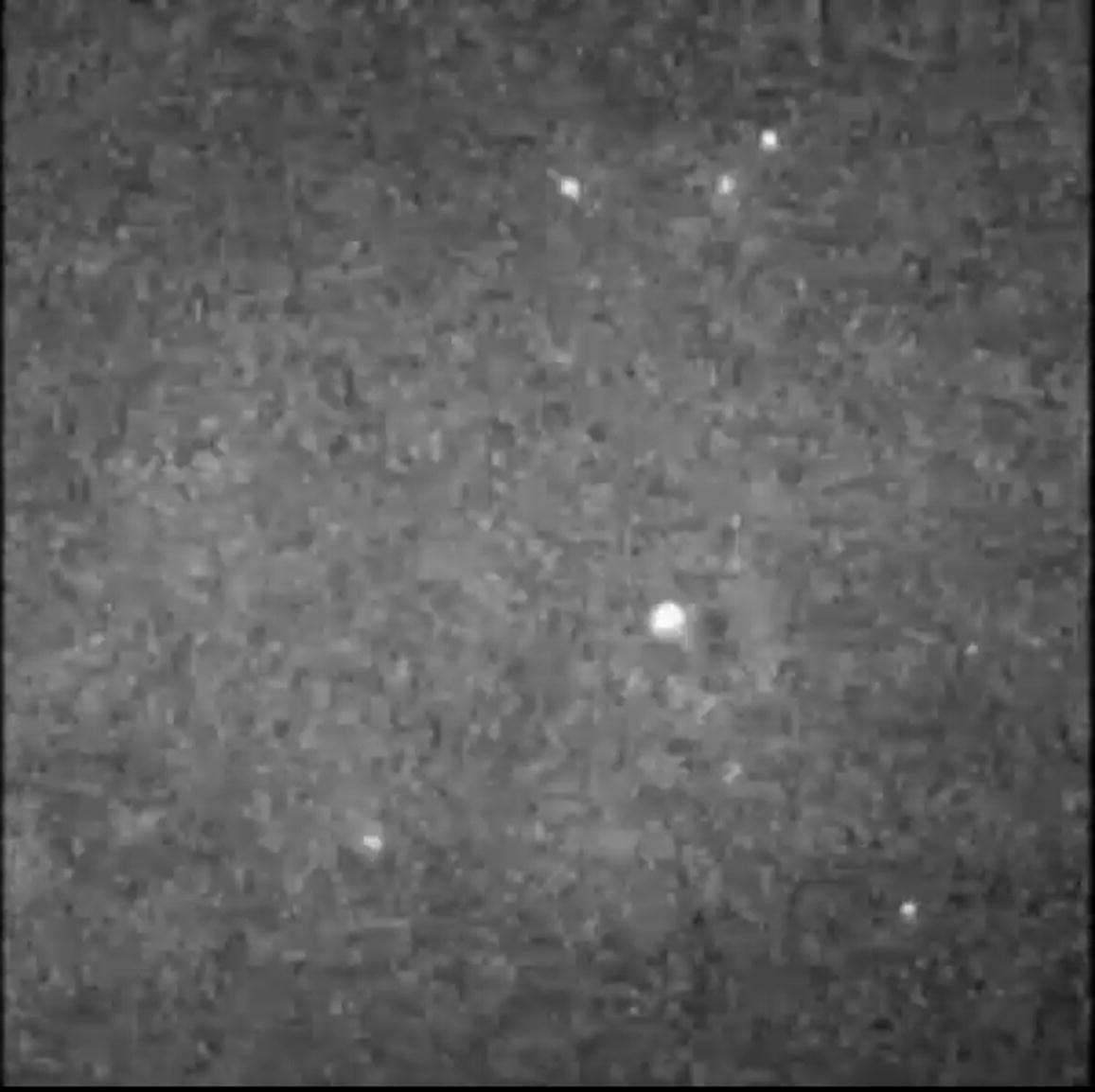

Supplement: Supplementary movie 9 — YFP blinking in an IRF section at atmospheric pressure acquired using a Nikon N-STORM microscope system. [file mmc9.jpg]
